# Effects of oral hygiene management containing *Cibotium Barometz J. Smith* extract on peri-implant mucositis: a randomized clinical trial

**DOI:** 10.1186/s12906-025-04900-3

**Published:** 2025-05-06

**Authors:** Yu-Rin Kim, Seoul-Hee Nam

**Affiliations:** 1 Department of Dental Hygiene, Silla University, 140 Baegyang-daero 700beon-gil, Sasang-gu, Busan, 46958 Republic of Korea; 2 Department of Dental Hygiene, College of Health Sciences, Kangwon National University, Samcheok, Gangwon-do 25945 Republic of Korea

**Keywords:** Biological products, Dental implnats, Oral health, Periodontitis, Toothbrushing

## Abstract

**Background:**

The purpose of this study was to confirm the improvement of peri-implant mucositis (PIM) in patients using the toothpick method (TPM) containing a natural ingredient: *Cibotium barometz J. Smith* (CB).

**Methods:**

As a randomized, blind, controlled trial study, there were 21 participants in the CB TPM group (CBG), 20 participants in the chlorhexidine group (CG), and 19 participants in the saline group (SG). All three groups were examined both before and after TPM. We performed a paired t-test to determine the differences between the mean values of the three groups. We used ANOVA to evaluate the mean differences in clinical indicators, saliva tests, and periodontal bacteria.

**Results:**

There were no significant differences in sociodemographic characteristics and oral health status between the three groups, thus ensuring homogeneity among the participants. Occult blood in the saliva test increased for SG and CG and decreased for CBG. Compared to the before-TPM condition, leukocyte and protein levels decreased for CG and CBG after TPM (*p* < 0.05). As a result of checking the effect size (Cohen’s d) before and after TPM according to the three groups, three species of bacteria were identified that showed a significant effect only on CBG, and two species were identified that showed a significant effect on CBG and CG (Cohen’s d ≥ 0.8).

**Conclusion:**

This study confirmed the efficacy of CB-applied TPM on PIM patients and demonstrated its potential as a non-surgical treatment option. Therefore, practitioners could extensively use CB-applied TPM as a mechanical tool and safe chemical and biological removal agent for post-implant management in clinical settings.

**Trial registration:**

The trial was registered in the International Clinical Trial Registry Platform (ICTRP) under the registration number KCT0008626 on 17/07/2023 and was retrospectively registered.

**Supplementary Information:**

The online version contains supplementary material available at 10.1186/s12906-025-04900-3.

## Background

Despite dental implants’ overall satisfactory survival rate, unsuccessful outcomes sometimes occur due to various inflammatory diseases in the peri-implant tissues [1]. Peri-implant diseases refer to an inflammatory process in the tissues surrounding the dental implants [2]. The prevalence of peri-implantitis has been reported to be as high as 56%, and the reported prevalence widely ranges from 12% to 43% [3]. Peri-implant mucositis (PIM) is defined as a reversible inflammatory process in the soft tissue surrounding the implant, whereas peri-implantitis involves additional loss of peri-implant bone [4]. Scholars generally view PIM as a precursor of peri-implantitis [5]. Therefore, prevention and management of PIM are essential in the long-term management of dental implants.

In animal and clinical studies, biofilm deposition on the implant surface has been identified as animportant etiological factor in initiating and developing peri-implant inflammation [6]. Several studies investigating the biofilm-related etiology of peri-implant inflammation have identified plaque removal as an essential step in resolving PIM [7]. Therefore, plaque removal from the peri-implant sites is considered critical in the long-term management of dental implants [8]. Physical oral hygiene practices that remove dental plaque, such as toothbrushing [9] or oral detergent [10], should be prioritized for plaque removal. A single or combination treatment of systemic antibiotics, such as penicillin and metronidazole, may be used 5 to 14 days after the practice [11]. Furthermore, 0.12% chlorhexidine can be used for oral cavity disinfection [10]. The surgical treatment method for PIM is to remove bacteria, their by-products, calculus, and soft tissue attached to the implant surface where the surrounding bone has been lost, thereby creating an environment in which bone re-adhesion can occur [12]. Therefore, dental plaque management is essential in preventing and treating PIM.

The toothpick method (TPM), in which an expert directly performs Watanabe toothbrushing using a double-row type medium bristle toothbrush, is an effective method for dental plaque removal [13] that efficiently prevents and improves PIM [14]. Chlorhexidine is effective when combined with TPM to treat PIM; its usage is gradually increasing [15]. However, prolonged usage of chlorhexidine stains the teeth and gingiva, changes taste perception, and increases supragingival calculus [16]. Furthermore, it can cause adverse epitheliolysis of oral mucosa when used in pediatric patients. Moreover, adverse cases of shock, hypersensitivity, and temporary parotitis have also been reported for the drug [10]. Additionally, when chlorhexidine is used in combination with other drugs (including toothpaste), cationic component biding can reduce its efficacy [17]. Thus, there is an increasing interest in replacing such chemical ingredients with natural substances showing bacterial inhibitory effects. Various studies have been conducted in this regard [18].

Natural substances with demonstrated therapeutic effects for PIM include *Plectranthus scutellarioides* (L.) R. Br. Leaves extract [19] and *Robusta Green Coffee Bean* (*Coffea Canephora*) [20]. Often used in traditional Asian medicine, *Cibotium barometz J. Smith* (CB) has been reported to have bioactive properties, such as anti-inflammatory effects in cells and bones. Various studies have been conducted to investigate CB’s therapeutic properties: antioxidation [21], anti-inflammation [22], hemostasis (post-tooth extraction) [23], regeneration and recovery of nerve cells [24], and osteoclast formation inhibition [25]. However, no study has been conducted to investigate the combinatory effects of TPM and CB for managing PIM. Therefore, we investigated the antibacterial properties of the natural extract of CB against PIM-related bacteria in PIM patients subjected to TPM. Furthermore, CB was compared against chlorhexidine and saline, which are frequently used with TPM for implant patients in the clinic, to demonstrate the utility of the substance.

## Methods

### Ethical consideration

As a human subject research, this study was conducted following the International Council for Harmonization of Technical Requirements for Pharmaceuticals for Human Use (ICH) guidelines. Research ethics were approved by Kangwon National University (KWNUIRB-2023-04-007-001, Chuncheon, South Korea). Furthermore, the WHO International Clinical Trial Registry Platform (ICTRP) (registration date: 17/07/2023; registration number: KCT0008626; https://cris.nih.go.kr/cris/search/detailSearch.do/24981) was retrospectively registered for clinical trial. This study was also conducted in accordance with the Consolidated Standards of Reporting Trials (CONSORT) guidelines [26]. The purpose and procedures of this study were described to all participants before commencing the study, and written consent was obtained from all subjects prior to inclusion.

### CB extraction

Dried CB purchased from Foodsynergy Co., Ltd. (Seoul, South Korea) was ground and mixed with 70% ethanol, followed by soaking and mixing at 60 °C for 12 h. Next, the CB extract was filtered using filter paper (Advantec No. 2, Tokyo, Japan) and concentrated using a rotary vacuum evaporator (N-1300E.V.S. EYELA Co., Tokyo, Japan). Next, the concentrate was lyophilized (Ilshin Lab Co., Yangju, South Korea) at -80 ℃ to obtain CB-extracted powder, which was stored at -20 °C until usage.

### Study participants

**Fig. 1:**
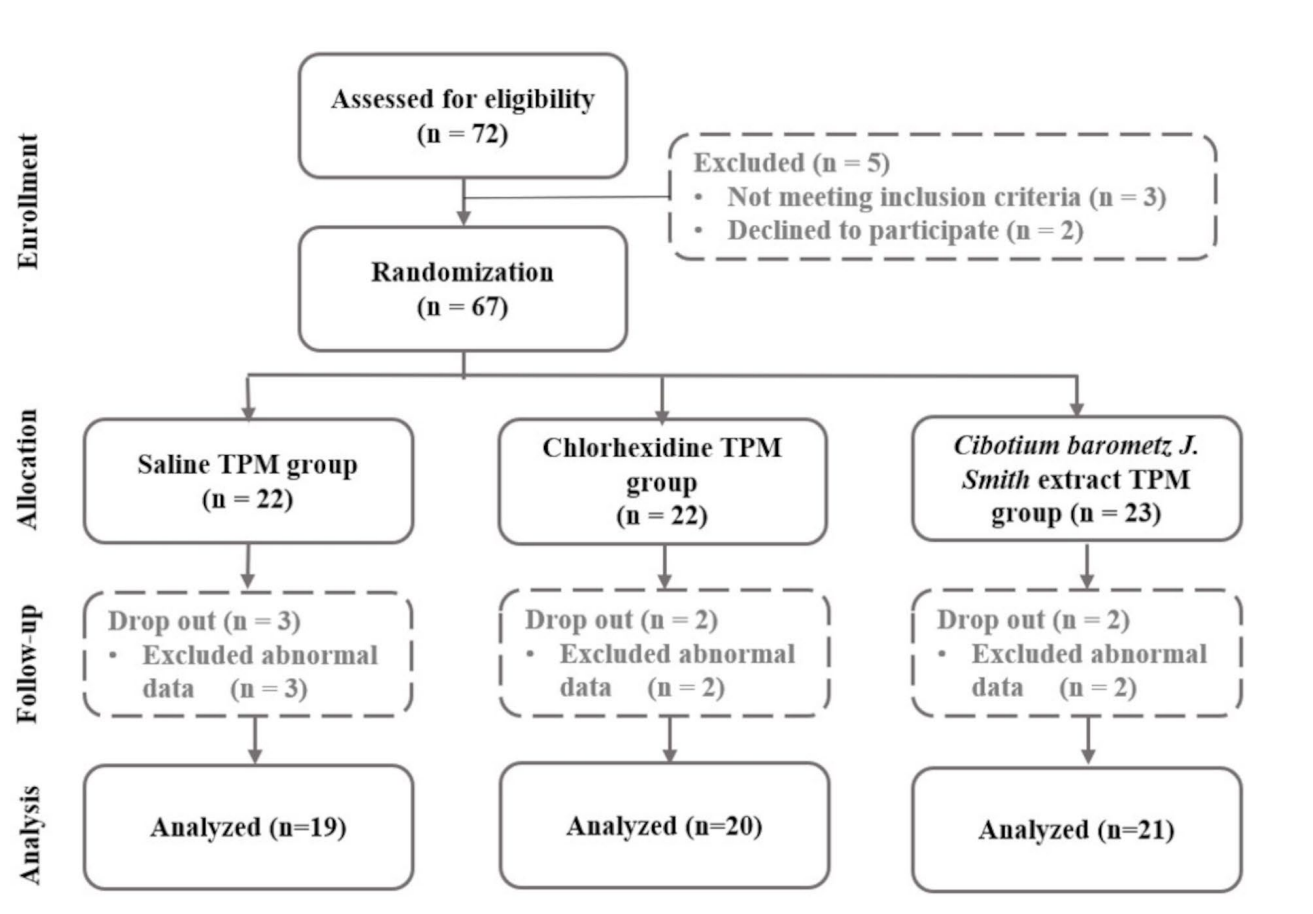
Flow chart of the study

The number of participants was determined using the G*Power 3.1 program [27] based on significance level a = 0.05 bilateral test, power = 0.95, and effect size = 0.5 [28]. The result indicated that 54 participants were required for the study. Prior to the study, the purpose, procedures, and risks of the study were explained to the participants. The participants were informed that they had the right to discontinue the study at any stage before the consent was given. Initially, there were 72 participants recruited for the study. After excluding five participants who declined to participate or did not meet the inclusion criteria, 67 participants were selected. These participants were then randomly divided into three groups according to the solutions. Participants were assigned using a computer-based randomization algorithm, using a computer-generated random number table. All process was kept confidential to both subjects and researchers, maintaining double-blindness. The participants who showed abnormal data (In case of missing values ​​or device measurement errors) were excluded from the study, ultimately resulting in 60 participants. There were 19 participants in the saline-TPM group (SG) as the negative control, 20 in the chlorhexidine-TPM group (CG) as the positive control, and 21 in the CB-TPM group (CBG) as the experimental group (Fig. 1).

### Study design and protocol

As a randomized blind, controlled trial study, the study participants were selected among the patients of “M Dental Clinic” located in Busan, South Korea between January and July 2023. A dental hygienist with more than ten years of experience explained the purpose of this clinical trial, and only those who gave consent were included in the study. The selection criteria for the subjects were as follows: over 20 years old, having at least one oral implant, and having been diagnosed with PIM within the past year. The diagnosis of PIM was based on a study by Papapanou et al. [29], and a dentist specializing in oral surgery diagnosed patients with inflammation limited to the soft tissue without bone loss around the implant. The exclusion criteria were as follows: having serious dental caries with at least one dentin caries, xerostomia, smoking habit, chronic diseases, current antibiotic treatment, severe periodontal diseases with at least one tooth with a periodontal pocket depth of more than 4 mm, periodontal surgery with gum incision within the past month, or bleeding disorders.

### Clinical examination

To ensure homogeneity in the oral environment among the participants, dental scaling was performed by a trained dental hygienist after an oral examination by a dentist. The study commenced after one week of recovery. Therefore, the study was conducted after removing dental calculus and plaque, which are clinical variables. Survey items included sociodemographic characteristics (age, gender, marriage, local disease not related to PIM), oral health status (oral examination, number of implants, subject satisfaction), The Periodontal Screening and Re-cording (PSR), and Bleeding of probing (BOP). As for the periodontal disease-related factors, saliva analysis and changes in 12 types of PIM-related bacteria were evaluated. TPM was performed using a double-row medium bristle toothbrush applied with 10 mg/mL CB extract (CBG), 0.12% chlorhexidine (CG), or saline (SG). After the scaling (one week before the study commencement), toothbrushes and toothpaste were provided to the participants. The participants were trained on the method and frequency of tooth brushing needed for the study. For all three groups, the clinical indicators related to the periodontal status were collected both before and after TPM. Data collection of all clinical indicators was performed by a single dental hygienist with more than 10 years of experience. In addition, to reduce intra-observer variation and increase agreement, PSR and BOP tests were conducted after sufficient practice one week before the start of the study.

### Variables

#### Demographic characteristics

As sociodemographic characteristics, gender and age of the participants were investigated. Furthermore, marital status was confirmed as married or single, and local diseases unrelated to PIM were investigated.

#### Oral health status

Oral examination, Number of implants, Subject satisfaction.

To investigate the oral health status of the participants, the signs of regular oral examination and the number of implants in the oral cavity were investigated. Furthermore, the level of satisfaction after TPM was surveyed for all three groups.

#### PSR

PSR is a modification of the Community Periodontal Index of Treatment Needs (CPITN), which was developed by the World Health Organization to estimate periodontal treatment needs in population groups [30]. With PSR, a specialized periodontal probe with a ball-shaped tip having a 0.5-mm diameter isused to examine and score six sites per tooth in each patient dentition sextant on a 0 to 4 hierarchical grading scale, identical to CPITN criteria,6with only the highest PSR score per sextant record for documentation. Sextants with only shallow probing depths (PDs) < 3.5 mm at all tooth sites are assigned a PSR index score of either 0, 1, or 2, depending on presence of BOP, dental calculus deposits, and/or defective dental restoration margins in the sextant. Sextants with deepest PDs ranging be-tween 3.5 and 5.5 mm are assigned a PSR index score of 3, whereas sextants with at least one > 5.5-mm PD receive a PSR index score of 4. In addition to the 0 to 4 grading scale, a code is to be added to PSR scores when additional periodontal abnormalities are detected in a dentition sextant, such as furcation involvements, excessive tooth mobility, mucogingival problems, and recession defects of ≥ 3.5 mm [31, 32].

#### BOP

BOP was measured using a standardized periodontal probe (CP11, Hu-Friedy Mfg. Co., LLC., Chicago, Illinois, USA) with 20–25 g probing force. One dental hygienist collected data after sufficient practice using a precision electronic scale (Precision Balances, ME-T Series, All4Lab Co., Ltd., Seoul, Korea) one week before the start of the study. The cases of bleeding within 30 s of probing the buccal and lingual surface of each tooth were recorded as a percentage. To determine the accuracy in measuring bleeding, the measurements were taken from the most posterior molar.

### Factors related to periodontal disease

#### Saliva test analysis system

According to the instructions provided for Sill-Ha ST-4910 (Arkray Inc., Kyoto, Japan) [33], the subjects rinsed their mouth with 3 mL of mouthwash for 10 s prior to spiting the saliva out into sterile tubes. The saliva was collected from the tube with a dropper and then placed on a measuring strip, which was then mounted on a measurement strip holder. Periodontal health (occult blood, leukocytes, proteins) was analyzed after 5 min and documented. The average values provided by the manufacturer were used as standard: average blood count = 22, average leucocyte count = 49, and average protein count = 43. It was evaluated that higher counts correlated with poorer periodontal health.

#### Microbiological analysis of PIM

To collect subgingival microbial samples, two sterilized paper points (size #15) were inserted into the area where the dental implant was placed for 10 s and then placed in a sterile 1.5 mL tube. Next, DNA was extracted from the samples using the AccuPrep Universal RNA Extraction Kit (Bioneer, Daejeon, South Korea) for microbial analysis. According to the manufacturer’s instruction, OligoMix (YD Global Life Science Co., Ltd., Seongnam, South Korea) and three oligonucleotides (forward primer, reverse primer, and probe), which react specifically to each bacterium, were used [34]. The 12 types of PIM-related bacteria (Table 1) were as follows: *Parvimonas micra (P. micra)*,* Eubacterium nodatumm (E. nodatumm)*,* Porphyromonas gingivalis (P. gingivalis)*,* Tannerella forsythia (T. forsythia)*,* Treponema denticola (T. denticola)*,* Fusobacterium nucleatum (F. nucleatum)*,* Prevotella intermedia (P. intermedia)*,* Prevotella nigrescens (P. nigrescens)*,* Eikenella corrodens (E. corrodens)*,* Campylobacter rectus (C. rectus)*,* Aggregatibacter actinomycetemcomitans (A. actinomycetemcomitans)*,* Staphylococcus aureus (S. aureus)* [35]. First, 9 µL of OligoMix, 10 µL of 2x probe qPCR mix (Takara Bio Inc., Shiga, Japan), and 1 µL of template DNA were mixed. Next, the polymerase chain reaction (PCR) reaction samples were placed in a 96-well plate, which was installed in the CFX96 Touch Real-Time PCR Detection System (Bio-Rad, Hercules, USA) for DNA segment amplification. The cycle condition of PCR was as follows: PCR initial activation step at 95 °C for 30 s, denaturation for 10 s at 95 °C, and annealing at 62 °C for 30 s with 40 repeated cycles. The result was then analyzed as cycle threshold (Ct) using Bio-Rad CFX Manager Software.

**Table 1:** Primers and probes used in the real-time PCR assays

Bacteria	Target genes	Primers/Probe sets	Amplicon size (bp)
*Parvimonas micra*	16 S ribosomal RNA gene	5′-GAGGAATACCGGTGGCGAAG-3’ 5′-GGCACCGAGATTTGACTCCC-3’ 5′-FAM-GGTACGAAAGCGTGGGGAGCA-BHQ1–3’	148
*Eubacterium nodatum*	hypothetical protein	5′-TGCTTGCCGGTGACTTAGGA-3’ 5′-AAACCGGGCTCAACAACCAT-3’ 5′-Texas Red-TTGAGGAGCCGGTGACTTTGG-BHQ2–3’	130
*Porphyromonas gingivalis*	hemagglutinin (phg) gene	5′-ACACGGTGTATCGTGACGGC-3’ 5′-GCCGGCTGCGTACTTAACCT-3’ 5′-HEX-CGACCTACCGCGATGCAGGA-BHQ1–3’	119
*Tannerella forsythia*	karilysin protease gene	5′-TGGCAAATCGCTCATCATCC-3’ 5′-TTCCATGTTCCCCAACCACA-3’ 5′-Texas Red-CCATTAAGCCCATTGCCCGG-BHQ2–3’	140
*Treponema denticola*	oligopeptidase B (opdB) gene	5′-AGAAAGGCTTTGGGCGACAG-3’ 5′-GCTGGAGCCGTAGCTTCCAT-3’ 5′-Cy5-CGGGTCCTCACCCGCTCTTC-BHQ2–3’	127
*Fusobacterium nucleatum*	16 S ribosomal RNA gene	5′-GGCTGTCGTCAGCTCGTGTC-3’ 5′-CTCATCGCAGGCAGTATCGC-3’ 5′-FAM-AACGAGCGCAACCCCTTTCG-BHQ1–3’	114
*Prevotella intermedia*	hemagglutinin (phg) gene	5′-CACACGCTGGCGAAACCTAC-3’ 5′-CACGTGGCGTTGCTTCTTTC-3’ 5′-HEX-CCGAAGATGCGCCGTTGAAC-BHQ1–3’	143
*Prevotella nigrescens*	gyrase subunit B (gyrB) gene	5′-AGCAAGCTGTAGGCGAGGCT-3’ 5′-GCTGAACACTTTCGCGTGCT-3’ 5′-Texas Red-GCTCGTATTGCAGCCCGCAA-BHQ2–3’	132
*Eikenella corrodens*	proline iminopeptidase (pip) gene	5′-GCCAACTGCTGCTGGAAGTG-3’ 5′-GCCGCTGATTTCGGAGAGTT-3’ 5′-HEX- ACAGCCATCGGCACAGGCAT-BHQ1–3’	110
*Campylobacter rectus*	groEL gene	5′-AAATTTAAGCGGCGACGAGG-3’ 5′-TCCTTGCTCACGCTTACGGA-3’ 5′-HEX-GGCTTTGACGCGGGCGTAGT-BHQ1–3’	132
*Aggregatibacter actinomycetemcomitans*	leukotoxin gene	5′-CGGGGCTTTCTACTACGGGA-3′ 5′-ATGCCTCAAGCATTCTCGCA-3′ 5′-FAM-GGTCAGCTTGGCAATCAGCCC-BHQ1–3’	123
*Staphylococcus aureus*	clumping factor A (clfA) gene	5′-GCGCAAGTAACGAAAGCAAAA-3’ 5′-GATTTTGCGCCACACTCGTT-3’ 5′-FAM-TGCTGCACCTAAAACAGACGACACA-BHQ1–3’	132

### Statistical analysis

All obtained clinical results were analyzed at a significance level of 5% using SPSS 24.0 for Windows (IBM Corp., Armonk, NY, USA). To confirm the difference in demographic characteristics and periodontal-related clinical indicators between SC, CG, and CBG, ANOVA and chi-square tests were conducted. Paired t-test was conducted to verify the differences in indicators between pre- and post-TPM for all three groups. To check the effect size of the difference before and after TPM, Cohen’s d was checked, and the effect size was divided into 0.2 (small effect size), 0.5 (medium effect size), and 0.8 (large effect size) [36] Furthermore, ANOVA was conducted to evaluate the average differences in saliva tests and PIM-related bacteria levels between the three groups. Duncan’s post-hoc test was performed for post hoc analysis. To confirm the effect sizes of the three groups, the η² values ​​were divided into 0.01 (small effect size), 0.06 (medium effect size), and 0.14 (large effect size) [36].

## Results

### Population characteristics

There were more females than males in SG and CG, whereas CBG was vice versa. As for the average age, SG was the lowest, and CBG was the highest. In all three groups, there were more married participants than unmarried participants and more participants without locoregional diseases than participants with locoregional diseases. There was no significant difference in sociodemographic characteristics between the three groups, ensuring homogeneity among the groups (Table 2).

**Table 2:** Characteristics of the subjects in SG, CG, and CBG

Characteristics		*N* (%)			*p*-value
		SG (*n* = 19)	CG (*n* = 20)	CBG (*n* = 21)	
^*^Gender	Male	8 (42.1)	8 (40.0)	12 (57.1)	0.525
	Female	11 (57.9)	12 (60.0)	9 (42.9)	
^¥^Age (mean ± SD)		58.05 ± 9.11	59.75 ± 8.45	61.43 ± 11.53	0.141
^*^Marriage	Single	3 (15.8)	3 (15.0)	3 (14.3)	1.000
	Married	16 (84.2)	17 (85.0)	18 (85.7)	
^*^Locoregional disease	No disease	14 (73.7)	13 (65.0)	13 (61.9)	0.742
	Have a disease	5 (26.3)	7 (35.0)	8 (38.1)	

^¥^*p*-values were determined by ANOVA test, ^*^p-values were determined by chi-square test, (*p* < 0.05); Values are presented as mean ± standard deviation

### Changes in oral health status

In all three groups, the ratio of those who received regular oral examinations was high compared to those who did not, and the number of dental implants in the mouth was lowest in CG and highest in SG. All three groups showed a higher proportion of code 2 in PSR. The BOP rate and number of implants were the highest in SG and lowest in CG. The homogeneity among the groups was ensured as there was no significant difference in the oral health status between the three groups. Furthermore, the level of satisfaction of the participants was 1 out of 5 for SG and CG, and 4 out of 5 for CBG. However, there was no significant difference (Table 3).

**Table 3:** Oral health status of the subjects in SG, CG, and CBG

Variables		*N* (%)			*p*-value
		SG (*n* = 19)	CG (*n* = 20)	CBG (*n* = 21)	
^*^Regular oral examination	Non-regular checkup	7 (36.8)	8 (40.0)	10 (47.6)	0.803
	Regular checkup	12 (63.2)	12 (60.0)	11 (52.4)	
^*^Periodontal screening and recording	Code 1	3 (15.8)	4 (20.0)	3 (14.3)	0.912
	Code 2	16 (84.2)	16 (80.0)	18 (85.7)	
^¥^Bleeding on probing (mean ± SD)		6.00 ± 6.07	5.50 ± 5.82	5.90 ± 7.28	0.967
^¥^Number of implants (mean ± SD)		11.68 ± 3.77	9.75 ± 2.17	10.52 ± 5.08	0.303
^¥^Satisfaction with disinfectant solution (mean ± SD)		1.58 ± 0.51	1.40 ± 0.50	4.43 ± 0.51	0.929

^¥^*p*-values were determined by ANOVA test, ^*^p-values were determined by chi-square test, (*p* < 0.05); Values are presented as mean ± standard deviation

### Periodontal related saliva analysis

Table 4 shows the saliva test results of the three groups. No meaningful difference was observed between the three groups before TPM. After TPM, there was a significant difference between the three groups in the occult blood (η² = 0.459, *p <* 0.001), protein (η² = 0.629, *p <* 0.001), and total counts (η² = 0.417, *p* < 0.001). Post hoc analysis of the post-TPM results indicated a clear difference between the three groups. After TPM, SG and CG showed increased levels of occult blood count, whereas CBG showed a significant decrease compared to pre-TPM levels (η² = 1.148, *p* < 0.001). All three groups showed decreased leukocyte and protein levels after TPM. However, significant differences between pre- and post-TPM values were only observed for CG (Leukocytes; Cohen’s d = 1.248, *p <* 0.001, Proteins; Cohen’s d = 0.599, *p* = 0.015) and CBG (Leukocytes; Cohen’s d = 1.462, *p <* 0.001, Proteins; Cohen’s d *=* 1.965, *p <* 0.001), with no significant difference shown for SG. In the case of Total, SG increased, while CG (Cohen’s d *=* 0.640, *p* = 0.010) and CBG (Cohen’s d *=* 2.281, *p* < 0.001) significantly decreased (Table 4).

**Table 4:** Saliva test results of the subjects in SG, CG, and CBG

Variables		Estimated Mean ± SE		Cohen’s d	^*^p-value
		Before TPM	After TPM		
Occult blood	SG	53.63 ± 0.45	77.26 ± 2.31^a^	-2.212	**< 0.001**
	CG	51.75 ± 3.35	52.10 ± 4.83^b^	-0.017	0.942
	CBG	55.19 ± 5.65	37.19 ± 4.50^c^	1.148	**< 0.001**
	η²	0.007	0.459		
	^¥^*p-*value	0.823	**< 0.001**		
Leukocytes	SG	60.00 ± 8.19	48.26 ± 6.66	0.382	0.114
	CG	61.50 ± 4.38	41.45 ± 3.56	1.248	**< 0.001**
	CBG	78.38 ± 6.45	37.05 ± 7.03	1.462	**< 0.001**
	η²	0.083	0.030		
	^¥^*p-*value	0.084	0.418		
Proteins	SG	48.63 ± 6.67	43.37 ± 3.10^a^	0.208	0.377
	CG	38.65 ± 4.79	24.10 ± 2.87^b^	0.599	**0.015**
	CBG	54.43 ± 4.91	9.05 ± 1.12^c^	1.965	**< 0.001**
	η²	0.072	0.629		
	^¥^*p-*value	0.119	**< 0.001**		
Total	SG	162.26 ± 14.52	168.89 ± 37.27^a^	-0.131	0.576
	CG	151.90 ± 8.11	117.65 ± 9.09^b^	0.640	**0.010**
	CBG	188.00 ± 12.31	83.29 ± 10.51^c^	2.281	**< 0.001**
	η²	0.080	0.417		
	^¥^*p-*value	0.094	**< 0.001**		

^¥^*p*-values were determined by ANOVA and Duncan’s post-hoc tests, ^*^p-values were determined by paired t-test (*p* < 0.05); Values are estimated mean ± standard error; significant (bold); different letters (a, b, and c) indicate statistically significant parameters

### PIM-related bacteria levels

PIM-related bacteria levels are shown in Table 5. No significant difference was observed between the three groups before TPM. After TPM, there was a significant difference between the three groups (*p* < 0.05). Comparative analysis between the groups after TPM indicated distinctly different bacteria levels for CBG compared to other groups, as *P. micra* (η² = 0.217, *p* < 0.001), *P. gingivalis* (η² = 0.086, *p* = 0.021), *E. nodatum* (η² = 0.135, *p* = 0.016), and *P. intermedia* (η² = 0.167, *p* = 0.006) were only identified in CBG. Furthermore, *T. forsythia* (η² = 0.180, *p* = 0.003), *T. denticola* (η² = 0.192, *p* = 0.002), *F. nucleatum* (η² = 0.193, *p* = 0.002), *P. nigrescens* (η² = 0.276, *p* < 0.001), *C. rectus* (η² = 0.204, *p* = 0.002), and *A. actinomycetemcomitans* (η² = 0.196, *p* = 0.002), *S. aureus* (η² = 0.292, *p* < 0.001) were identified in CG and CBG, indicating their similar efficacy. Additionally, in *E. corrodens* (η² = 0.117, *p* = 0.029), CBG and CG were classified into the same group and had similar reduction effects. Comparison between pre- and post-TPM levels for each group indicated a significant increase in the *P. micra* (SG; Cohen’s d = -0.731, *p =* 0.005, CG; Cohen’s d = -0.696, *p =* 0.006, CBG; Cohen’s d = 1.088, *p* < 0.001) and *E. nodatum* (SG; Cohen’s d = -0.577, *p =* 0.022, CG; Cohen’s d = -0.626, *p =* 0.011, CBG; Cohen’s d = 0.632, *p* = 0.009) levels after TPM for SG and CG, whereas a significant decrease was observed for CBG. A significant increase in SG (Cohen’s d = -0.497, *p* = 0.044) and decreased CBG (Cohen’s d = 0.631, *p* = 0.009) were observed in *T. forsythia* after TPM. Furthermore, CG (Cohen’s d = -0.630, *p* = 0.011) showed a significant increase in the *T. denticola* level after TPM. Notably, only CG and CBG showed a significant decrease in the *F. nucleatum* (CG; Cohen’s d = 1.832, *p* < 0.001, CBG; Cohen’s d = 2.007, *p* < 0.001,), *P. nigrescens* (CG; Cohen’s d = 0.563, *p =* 0.021, CBG; Cohen’s d = 4.055, *p* < 0.001), and *S. aureus* (CG; Cohen’s d = 0.907, *p <* 0.001, CBG; Cohen’s d = 1.122, *p* < 0.001) levels after TPM. Only CBG (Cohen’s d = 0.912, *p* < 0.001) decreased significantly in *P. intermedia* post TPM. In all three groups, the levels of *E. corrodens* (SG; Cohen’s d = 0.504, *p =* 0.041, CG; Cohen’s d = 0.557, *p =* 0.022, CBG; Cohen’s d = 0.508, *p* = 0.030) and *A. actinomycetemcomitans* (SG; Cohen’s d = 0.582 *p =* 0.021, CG; Cohen’s d = 0.563, *p =* 0.021, CBG; Cohen’s d = 0.553, *p* = 0.020) showed significant decreases after TPM. Additionally, *C. rectus* showed a significant increase for SG (Cohen’s d = -0.590, *p* = 0.019) after TPM but showed a significant decrease for CBG (Cohen’s d = 0.715, *p* = 0.004) (Table 5).

**Table 5:** Differences in periodontal disease-causing oral bacteria according to gargel application

Variables		Estimated Mean ± SE		Cohen’s d	^*^p-value
		Before TPM	After TPM		
*Parvimonas micra*	SG	110.42 ± 49.49	886.89 ± 227.07^a^	-0.731	**0.005**
	CG	128.35 ± 27.54	560.50 ± 163.56^a^	-0.696	**0.006**
	CBG	129.62 ± 21.19	21.62 ± 5.08^b^	1.088	**< 0.001**
	η²	0.003	0.217		
	^¥^*p-*value	0.907	**< 0.001**		
*Eubacterium nodatum*	SG	90.74 ± 32.11	243.47 ± 92.76^a^	-0.577	**0.022**
	CG	93.20 ± 17.92	312.15 ± 89.93^a^	-0.626	**0.011**
	CBG	91.00 ± 25.10	22.19 ± 8.46^b^	0.632	**0.009**
	η²	0.000	0.135		
	^¥^*p-*value	0.997	**0.016**		
*Porphyromonas gingivalis*	SG	725.21 ± 267.52	646.74 ± 144.62^a^	0.080	0.732
	CG	742.15 ± 75.47	607.15 ± 261.17^a^	0.117	0.606
	CBG	771.62 ± 472.40	32.57 ± 10.82^b^	0.344	0.131
	η²	0.056	0.086		
	^¥^*p-*value	0.995	**0.021**		
*Tannerella forsythia*	SG	4399.63 ± 936.22	23930.63 ± 9268.98^a^	-0.497	**0.044**
	CG	5205.45 ± 1073.75	3280.90 ± 1025.74^b^	0.215	0.347
	CBG	5238.43 ± 1776.62	79.24 ± 36.53^b^	0.631	**0.009**
	η²	0.004	0.180		
	^¥^*p-*value	0.886	**0.003**		
*Treponema denticola*	SG	6180.79 ± 2857.11	12511.00 ± 4855.97^a^	-0.302	0.195
	CG	494.90 ± 150.71	594.30 ± 185.80^b^	-0.630	**0.011**
	CBG	3395.00 ± 1834.77	127.33 ± 54.65^b^	0.386	0.092
	η²	0.070	0.192		
	^¥^*p-*value	0.128	**0.002**		
*Fusobacterium nucleatum*	SG	590103.84 ± 33348.88	415487.32 ± 146436.67^a^	0.302	0.204
	CG	584661.40 ± 56272.09	93566.15 ± 25681.43^b^	1.832	**< 0.001**
	CBG	551931.62 ± 58480.72	8349.43 ± 1862.00^b^	2.007	**< 0.001**
	η²	0.006	0.193		
	^¥^*p-*value	0.849	**0.002**		
*Prevotella intermedia*	SG	3779.95 ± 321.86	5069.16 ± 1485.80^a^	-0.207	0.378
	CG	3789.45 ± 269.82	6638.45 ± 1985.79^a^	-0.288	0.214
	CBG	3861.14 ± 861.84	273.90 ± 96.03^b^	0.912	**< 0.001**
	η²	0.000	0.167		
	^¥^*p-*value	0.994	**0.006**		
*Prevotella nigrescens*	SG	745.53 ± 332.76	1068.37 ± 273.25^a^	-0.157	0.503
	CG	741.70 ± 164.09	339.70 ± 105.44^b^	0.563	**0.021**
	CBG	736.14 ± 39.50	21.05 ± 7.14^b^	4.055	**< 0.001**
	η²	0.000	0.276		
	^¥^*p-*value	0.999	**< 0.001**		
*Eikenella corrodens*	SG	157.37 ± 48.96	39.47 ± 11.19^a^	0.504	**0.041**
	CG	150.00 ± 49.47	25.00 ± 9.93^ab^	0.557	**0.022**
	CBG	150.86 ± 64.64	6.38 ± 2.60^b^	0.508	**0.030**
	η²	0.000	0.117		
	^¥^*p-*value	0.995	**0.029**		
*Campylobacter rectus*	SG	100.63 ± 22.59	2363.00 ± 886.70^a^	-0.590	**0.019**
	CG	110.80 ± 23.07	115.50 ± 36.11^b^	-0.020	0.928
	CBG	104.90 ± 28.95	8.29 ± 3.56^b^	0.715	**0.004**
	η²	0.001	0.204		
	^¥^*p-*value	0.961	**0.002**		
*Aggregatibacter actinomycetemcomitans*	SG	346.84 ± 136.79	20.26 ± 7.99^a^	0.582	**0.021**
	CG	329.50 ± 130.93	0.00 ± 0.00^b^	0.563	**0.021**
	CBG	317.00 ± 125.17	0.00 ± 0.00^b^	0.553	**0.020**
	η²	0.000	0.196		
	^¥^*p-*value	0.987	**0.002**		
*Staphylococcus aureus*	SG	47.47 ± 8.23	174.68 ± 50.49^a^	-0.583	**0.020**
	CG	44.30 ± 6.58	10.00 ± 4.59^b^	0.907	**< 0.001**
	CBG	45.67 ± 6.87	4.95 ± 2.28^b^	1.122	**< 0.001**
	η²	0.002	0.292		
	^¥^*p-*value	0.954	**< 0.001**		

^¥^*p*-values were determined by ANOVA and Duncan’s post-hoc tests, ^*^p-values were determined by paired t-test (*p* < 0.05); Values are estimated mean ± standard error; significant (bold); different letters (a and b) indicate statistically significant parameters

## Discussion

Dental implants have been widely used over the past decade to replace damaged teeth, demonstrating a high success rate of 94.6% [37]. Despite this remarkable success, 10–40% of cases show side effects like PIM [38]. The abutment area connected to the upper prosthesis is smaller than the natural teeth due to the implant structure, resulting in a large gap between the teeth. This makes the removal of biofilm difficult with simple toothbrushing. When the dental plaque is not properly removed, inflammation can occur in the surrounding tissues, leading to alveolar bone loss.

In terms of professional implant management, dental floss is reported to be a popular choice (76.3%). However, due to the rough surface of implants and dental floss fragments potentially caught in the sulcus around the implant, dental floss can cause and contribute to peri-implant diseases [39, 40]. Therefore, TPM has been widely used for implant management in dental clinical practice, showing notable efficacy [41]. The satisfaction level for TPM among the patients is rated at 4.6 out of 5.0, with 92.5% of the patients expressing an intention to receive the treatment again in the future. Numerous studies have reviewed the effects of TPM against PIM and explored the benefits of chlorhexidine applied with TPM. There are fewer studies on natural alternatives to synthetic chemicals. Therefore, we compared the efficacy of the CB extract, which is a well-known natural alternative with anti-inflammatory properties, against chlorhexidine and saline applied with TPM.

The homogeneity among the test groups (CBG, CG, and SG) was ensured as no significant differences in sociodemographic characteristics, oral health status, oral environment, and bacteria were observed between the groups. The results indicated that the salivary occult blood count increased for CG and SG after TPM, but decreased for CBG. The occult blood count in saliva effectively reflects and is recognized as an early symptom of periodontal disease [42]. Therefore, we claim that CB extract applied with TPM may reduce peri-implant inflammatory bleeding more efficiently than chlorhexidine or saline applied with TPM. Furthermore, the leucocyte and protein counts in the saliva are used as biomarkers of inflammation. Compared to patients with chronic periodontitis, patients with aggressive periodontitis had levels of C-reactive protein that were on average more than 50% higher [43]. Leucocyte increases in the presence of bacteria, which causes periodontal diseases. The results indicated that CB extract applied with TPM reduced the levels of protein and leucocyte more effectively than the effect observed with chlorhexidine applied with TPM. As a result of checking the effect size (Cohen’s d) before and after TPM according to the three groups, in the case of leukocytes, CG and CBG decreased to a similar effect size, while in the case of Occult blood, Proteins, and Total, only CBG decreased to a large effect size (Cohen’s d ≥ 0.8). As previously mentioned, chlorhexidine is frequently used with TPM for the management of implants [15]. Therefore, CB extract’s comparable efficacy may be beneficial for post-implant management, as it does not possess the side effects of synthetic chemical agents.

It is known that stable implants in healthy periodontal tissues are mainly colonized by cocci, similar to natural teeth in healthy periodontal tissues. In peri-impactitis lesions, cocci are rarely detected, while high proportions of spindle bacteria, motile bacilli, and spiral bacteria are found [44]. In particular, a relatively high ratio of red complex (*P. gingivalis*,* T. forsythia*,* T. denticola*) has been detected in these lesions [45]. In a study using checkerboard DNA-DNA hybridization, periodontal pathogens such as *P. gingivalis*,* P. intermedia*,* T. forsythia*,* A. actinomycetemcomitans*,* T. denticola* were found in patients with implantitis at high levels [46]. Furthermore, recent studies have reported a high level of *S. aureus* in peri-implantitis lesions [47]. Based on these findings, we investigated 12 types of oral bacteria in this study. The results indicated that CB extract and chlorhexidine, each applied with TPM, similarly decreased the levels of *T. forsythia*,* T. denticola*,* F. nucleatum*,* P. nigrescens*,* C. rectus*,* A. actinomycetemcomitans*, and *S. aureus*. Notably, CB extract decreased the levels of *P. micra*,* P. gingivalis*,* E. nodatum*, and *P. intermedia* more effectively than chlorhexidine. Chlorhexidine is known to cause cell death at a low concentration of 0.002% while inducing fibroblast necrosis at a high concentration [48]. Therefore, our results indicated that CB may be used as a safe alternative to chlorohexidine against PIM-related bacteria without causing side effects.

In a bacteria culture study by Bismelah and colleagues [19], *Prostanthera scutellarioides* (*P. scutellariaides)* was used as a natural antibacterial treatment against PIM. The treatment decreased the levels of *S. sanguinis*, *S. mitis*, *S. oralis*, *S. salivarius*, *A. actinomycetemcomitans*, *T. forsythia*,* P. gingivalis*, and *P. intermedia.* Here, *P. scutellariaides* showed a relatively low efficacy against *T. denticola.* In this study, CG and SG showed an increased level of *T. denticola*, while CBG effectively decreased the level. This is significant, as our study was a human-involved clinical trial, whereas the study by Bismelah and colleagues. was based on bacteria culture. So far, no study has demonstrated the efficacy of a natural alternative applied with TPM against PIM-related bacteria. Therefore, TPM in combination with anti-inflammatory natural compounds will continue to be evaluated in the clinic, along with in vitro verification. The results of this study indicated that SG significantly increased the levels of 5 (*P. micra*,* E. nodatum*,* T. forsythia*,* C. rectus*,* S. aureus*) out of 12 types of bacteria; CG increased the levels of 3 (*P. micra*,* E. nodatum*,* T. denticola*) out of 12 types of bacteria. Saline lacks intrinsic antibacterial properties, which may allow bacterial regrowth over time after cleansing [49]. Additionally, some studies have reported that bacteria surviving chlorhexidine treatment may proliferate more rapidly. Similarly, this study also observed an increase in certain bacterial counts in the SG and CG.

As a result of checking the effect size (Cohen’s d) before and after TPM according to the three groups, the bacteria that showed a great effect only on CBG were *P. micra*,* P. intermedia*, and *P. nigrescens*, and the bacteria that showed a great effect on CBG and CG were *F. nucleatum* and *S. aureus* (Cohen’s d ≥ 0.8). CB extract contains physiologically active substances (such as flavonoids, tannins, and terpenoids), which can respond to a wide range of pathogens through multiple antibacterial mechanisms, by destroying bacterial cell walls and cell membranes and affecting protein and enzyme activities to inhibit bacterial survival [22]. In contrast to chlorhexidine, which relies on a specific mechanism of action, CB extract is likely to act on multiple antibacterial pathways simultaneously, so it is thought to have a greater effect on reducing bactericidal activity across the range of effects. This result demonstrated the efficacy of CB extract in reducing harmful PIM-related bacteria. Nevertheless, the increased levels of some types of bacteria after TPM may be a limitation of this study. This may be attributed to the different topography of implant surfaces compared to the natural teeth surface, as well as the unique immunological characteristics and differences in responses of peri-implant tissues compared to the natural periodontal tissues. Furthermore, although inserted in the gingival groove of the same tooth, the bacterial deposition may not have been equal among the patients due to the potential differences in the oral environment and bacterial outflowing secretions. Despite these limitations, TPM is still an excellent non-surgical treatment option. When TPM is combined with CB extract, the treatment becomes synergistic through both mechanical and chemical removal of harmful bacteria. Based on our clinical investigation of CB extract’s excellent antibacterial properties, we claim its utility for the prevention and treatment of PIM. Considering the positive results of this study, further studies on the utility of other natural ingredients are warranted for practical and effective post-implant management.

## Conclusion

In this study, the therapeutic potential of CB extract in combination with TPM was tested for the non-surgical management of PIM patients. As a natural alternative to synthetic chemicals, CB extract may have clinical utility for not only PIM patients but also for those with implants as part of a post-implant management program. In order to confirm the applicability in various clinical settings, additional research including long-term follow-up studies and large-scale randomized controlled trials will be conducted in the future to more clearly identify the sustainability, safety, and effects in various patient groups.

## Electronic supplementary material

Below is the link to the electronic supplementary material.

Supplementary Material 1

Supplementary Material 2

Supplementary Material 3

Supplementary Material 4

## Data Availability

The data sets generated and/or analyzed during the current study are not publicly available for reasons of personal and organizational integrity but are available from the corresponding author on reasonable request.

## Abbreviations

PTM: Peri-implant mucositis
TPM: Toothpick method
CB: Cibotium barometz J. Smith
PSR: Periodontal Screening and Re-cording
CPITN: Community Periodontal Index of Treatment Needs
PDs: Probing depths
BOP: Bleeding on probing
PCR: Polymerase chain reaction
Ct: Cycle threshold
P. micra: Parvimonas micra
E. nodatumm: Eubacterium nodatumm
P. gingivalis: Porphyromonas gingivalis
T. forsythia: Tannerella forsythia
T. denticola: Treponema denticola
F. nucleatum: Fusobacterium nucleatum
P. intermedia: Prevotella intermedia
P. nigrescens: Prevotella nigrescens
E. corrodens: Eikenella corrodens
C. rectus: Campylobacter rectus
A. actinomycetemcomitans: Aggregatibacter actinomycetemcomitans
S. aureus: Staphylococcus aureus
P. scutellariaides: Prostanthera scutellarioides
