# Supplementary material for: Effects of oral hygiene management containing Cibotium Barometz J. Smith extract on peri-implant mucositis: a randomized clinical trial
Source: BMC Complement Med Ther. 2025 May 6;25:164. doi: 10.1186/s12906-025-04900-3 (PMC12054295; doi:10.1186/s12906-025-04900-3)
Supplement: Supplementary file 1 — Supplementary Material 1 [file 12906_2025_4900_MOESM1_ESM.pdf]

<Oral Examination>

ID		Age		sex	M/F

<Base>

Buccal																
Lingual																
Dental	8	7	6	5	4	3	2	1	1	2	3	4	5	6	7	8
Lingual																
Buccal																

PI	Right	Ant.	Left	GI	Right	Ant.	Left	BB checker<br>1st:<br>post:<br>3rd:
Upper				Upper				
Lower				Lower				

<Post of saline>

Buccal																
Lingual																
Dental	8	7	6	5	4	3	2	1	1	2	3	4	5	6	7	8
Lingual																
Buccal																

PI	Right	Ant.	Left	GI	Right	Ant.	Left	Cariview<br>1st:<br>post:<br>3rd:
Upper				Upper				
Lower				Lower				

<Post of G>

Buccal																
Lingual																
Dental	8	7	6	5	4	3	2	1	1	2	3	4	5	6	7	8
Lingual																
Buccal																

PI	Right	Ant.	Left	GI	Right	Ant.	Left	
Upper				Upper				
Lower				Lower				

<Post of 5days>

Buccal																
Lingual																
Dental	8	7	6	5	4	3	2	1	1	2	3	4	5	6	7	8
Lingual																
Buccal																

PI	Right	Ant.	Left	GI	Right	Ant.	Left	
Upper				Upper				
Lower				Lower				

Changes in oral environment according to the antibacterial effect of natural detergent extract

This is a question about demographic characteristics.

1. Gender	① Male ② Female
2. Age	( ) years
3. Systemic disease	① No disease ② High blood pressure ③ diabetes ④ Etc ( )
4. Marrige	① Single ② Married ③ Etc ( )

This is a question about oral health behavior.

1. How many times a day do you brush your teeth?

① Rarely (once every 2-3 days) ② 1 ③ 2 ④ 3 ⑤ 4<

2. When do you usually brush your teeth (check all that apply)

	Do	Don't
1) Immediately after waking up in the morning (before breakfast)	①	⓪
2) After breakfast	①	⓪
3) After lunch	①	⓪
4) After dinner	①	⓪
5) Before sleeping	①	⓪

This is a question about oral health.

Please circle (v) the question that you think is closest to how you felt in the past two months.

Questionnaire	At all no	No	Usu ally	Well	Very well
I feel that my overall oral health is healthy.					
There have been times when it was difficult for me to chew food because of problems with my teeth or mouth.					
I have had swollen or bleeding gums.					
Your gums bleed when you brush your teeth.					
I can't eat cold or hot food properly.					
You feel your mouth often dry (dry mouth).					
I feel bad breath (bad breath)					
I feel that my pronunciation is incorrect.					
There were times when there was a sound or pain in the jaw joint, or the mouth was not open well.					
I used to worry about my oral health.					

This is a question about bad breath.

Questions about oral health Do you feel bad breath on your own?
① Yes ⓪ No.

No bad breath (0)	Suspicious Smell (1)	Slightly bad breath(2)	Moderate bad breath (3)	Strong bad breath(4)	Serious bad breath(5)

Do you feel that your bad breath has improved by participating in this study?

① Yes ⓪ No
How do you feel about the improvement of bad breath through participation in this study?

Very good(5)	Good(4)	Usually(3)	Not really(2)	at all(1)
