# Supplementary material for: Effects of oral hygiene management containing Cibotium Barometz J. Smith extract on peri-implant mucositis: a randomized clinical trial
Source: BMC Complement Med Ther. 2025 May 6;25:164. doi: 10.1186/s12906-025-04900-3 (PMC12054295; doi:10.1186/s12906-025-04900-3)
Supplement: Supplementary file 2 — Supplementary Material 2 [file 12906_2025_4900_MOESM2_ESM.docx]

**Instructions and consent form for study participants**

Research project name: Anti-inflammatory effects of oral hygiene management containing Cibotium Barometz J. Smith extract on peri-implant mucositis: A randomized clinical trial

**Research Director Name:** Nam Seoul-hee (Associate Professor)

This study is a study on changes in the oral environment according to the antibacterial effect of *Lespedeza cuneata*, a natural extract against oral disease-causing bacteria. You were invited to participate in this study as a healthy person. Seol-Hee Nam, Research Director (033-540-3394) at Kangwon National University, who conducts this study, will explain this study to you. This study will be conducted only for those who have voluntarily expressed their intention to participate, and it is important for you to understand why this study is being conducted and what it relates to before deciding to participate. Please read the following carefully before deciding to participate and, if necessary, discuss it with your family and friends. If you have any questions, your researcher will explain in detail.

**1. Why is this study being conducted?**

The purpose of this study is to evaluate the possibility of using licorice extract (*Cibotium Barometz J. Smith*), a natural medicinal plant, as a natural drug for oral diseases by confirming its antibacterial effect on oral diseases.

**2. How many people participate?**

In this study, 82 people with healthy bodies will participate.

**3. If you participate in the study, what is the process?**

If you indicate your intention to participate, the following process will proceed.

You will receive a three-minute questionnaire at the Busan Miso Plant Dental Clinic in South Korea. After filling out simple personal information about gender and age, you will be given an oral examination. A bad breath test is performed as an oral examination item, and a microbial activity test, and a dental caries activity test are performed, which takes about 30 minutes.

**4. How long is the study participation period?**

The questionnaire and oral examination will take about 35 minutes and will all be completed on the same day.

**5. Can I stop participating midway through?**

You may withdraw from participation at any time and without any penalty.

If you wish to stop participating in the study, please inform the researcher immediately.

In addition, we will promptly destroy data collected in the study when you discontinue participation.

**6. Are there any side effects or risk factors?**

You may experience uncomfortable situations while undergoing an oral examination. In this case, you can stop the oral examination at any time. If you have any questions about side effects or risk factors that may occur while participating in the study, please contact the researcher immediately.

**7. Are there any benefits for participants in participating in this study?**

There is no direct benefit for you to participate in this study, and there is no monetary compensation of any kind. However, the information you provide will help us to better understand the efficacy of *Cibotium Barometz J. Smith* extract for oral diseases.

**8. Are there any disadvantages if I do not participate in this study?**

You are free to not participate in this study. In addition, there will be no disadvantage to you if you do not participate in this study.

**9. Is the confidentiality of all personal information obtained from research guaranteed?**

The person in charge of personal information management is Professor Nam Seoul-Hee of Kangwon National University located in South Korea, 82-33-540-3394. We will do our best to ensure the confidentiality of any personal information obtained through this research. Your name and other personal information will not be used when the personal information obtained from this research is disclosed in a scientific journal or conference. However, if required by law, your personal information may be provided. In addition, monitor agents, inspectors, and bioethics committees may directly view the research results to verify the reliability of the procedures and data of this research within the scope stipulated by the relevant regulations without infringing on the confidentiality of research participants' personal information. There is. By signing this consent form, you agree that you have been informed of and agree to do so.

**10. Will I be paid for participating in this study?**

There is no financial compensation to the research subjects for participating in this study.

**11. How can I contact my research inquiry?**

If you have any questions about this study or if you have any problems during the study, please contact the following research staff:

Name: Nam Seoul-Hee Phone number: 82-33-540-3394

If you have any questions about your rights as a research participant at any time, please contact the following Kangwon National University Institutional Review Board.

Kangwon National University Institutional Review Board

Phone number: 82-33-250-7905

**Agreement**

1. I have read this manual and discussed it with the researcher in charge.

2. I have heard about the risks and benefits and have received satisfactory answers to my questions.

3. I voluntarily consent to participate in this study.

4. I agree to the researcher's collection and processing of the information about me obtained in this study to the extent permitted by current laws and regulations of the Bioethics Committee.

5. When the researcher or an authorized representative conducts research or manages the results, and when the health authorities, school authorities, and Kangwon National University Bioethics Committee conduct a fact-finding investigation, I directly access my personally identifiable information that is kept confidential agree to do

6. I know that I may withdraw from this study at any time and that this decision will not harm me in any way.

7. My signature indicates that I have received a copy of this consent form, and I will keep a copy until the end of my participation in the study.

Name of research participant Signature Date (year/month/day)

Name of researcher

who received consent form Signature Date (year/month/day)

Research Director Name Signature Date (year/month/day)

If there is

Legal Representative Name

(Relationship to Participant) Signature Date (year/month/day)
