# Supplementary material for: Effects of oral hygiene management containing Cibotium Barometz J. Smith extract on peri-implant mucositis: a randomized clinical trial
Source: BMC Complement Med Ther. 2025 May 6;25:164. doi: 10.1186/s12906-025-04900-3 (PMC12054295; doi:10.1186/s12906-025-04900-3)
Supplement: Supplementary file 3 — Supplementary Material 3 [file 12906_2025_4900_MOESM3_ESM.pdf]

Research protocol: part 1

Project summary

Oral disease is not recoverable once it occurs [1], so prevention is more important than anything

Background: The purpose of this study was to confirm the improvement of peri-implant mucositis (PIM) in patients using the toothpick method (TPM) containing a natural ingredient: *Cibotium barometz J. Smith* (CB).

Methods: As a randomized, blind, controlled trial study, there were 21 participants in the *Cibotium barometz J. Smith* TPM group (CBG), 20 participants in the chlorhexidine group (CG), and 19 participants in the saline group (SG). To ensure the homogeneity of the oral environment among participants, we performed scaling one week before the experiment. All three groups were examined both before and after TPM. We performed a paired t-test to determine the differences between the mean values of the three groups. We used ANOVA to evaluate the mean differences in clinical indicators, saliva tests, and periodontal bacteria.

Results: There were no significant differences in sociodemographic characteristics and oral health status between the three groups, thus ensuring homogeneity among the participants. Occult blood in the saliva test increased for SG and CG and decreased for CBG. Compared to the before-TPM condition, leukocyte and protein levels decreased for CG and CBG after TPM ($p < 0.05$). Bacteria levels decreased for all groups after TPM: two types of bacteria in SG, five in GC, and ten in CBG ($p < 0.05$).

Conclusion: This study confirmed the efficacy of CB-applied TPM on PIM patients and demonstrated its potential as a non-surgical treatment option. Therefore, practitioners could extensively use CB-applied TPM as a mechanical tool and safe chemical and biological removal agent for post-implant management in clinical settings.

References

Ainamo, J., Barmes, D., Beagrie, G., Cutress, T., Martin, J., & Sardo–Infirri, J. (1982). Development of the World Health Organization (WHO) community periodontal index of treatment needs (CPITN). *Int. Dent. J., 32,* 281–291.

American Dental Association & American Academy of Periodontology. (2001). *Periodontal screening and recording—An early detection system* (pp. 1–8). Chicago: American Dental Association.

General information

1. Protocol Title: Anti-inflammatory effects of oral hygiene management containing Cibotium Barometz J. Smith extract on peri-implant mucositis: A randomized clinical trial

2. protocol identifying number: Kangwon National University (KWNUIRB-2023-04-007-001, Chuncheon, South Korea). Furthermore, the WHO International Clinical Trial Registry Platform

(ICTRP) (registration date: 17/07/2023; registration number: KCT0008626; https://cris.nih.go.kr/cris/search/detailSearch.do/24981) was used for clinical trial registration.

4. researchers

4.1 Principal Investigator: Department of Dental Hygiene, College of Health Sciences, Kangwon National University, Seoul-Hee Nam (Associate professor)

4.2. Co-researcher: Department of Dental Hygiene, Silla University, Yu-Rin Kim (Assistant professor)

5. Name(s) and address(es) of the clinical laboratory (ies) and other medical and/or technical department(s) and/or institutions involved in the research: Miso Plant Dental Clinic in South Korea. / 304, Suyeong-ro, Nam-gu, Busan/ 010.6686.8130

Rationale & background information

Despite dental implants' overall satisfactory survival rate, unsuccessful outcomes sometimes occur due to various inflammatory diseases in the peri-implant tissues (Berglundh, Persson & Klinge, 2002). Peri-implant diseases refer to an inflammatory process in the tissues surrounding the dental implants (Zitzmann & Berglunch, 2008). The average prevalence of peri-implant mucositis (PIM) and peri-implantitis are 43% and 22%, respectively (Derks & Tomasi, 2015). PIM is defined as a reversible inflammatory process in the soft tissue surrounding the implant, whereas peri-implantitis involves additional loss of peri-implant bone (Lang, Bragger, Walther, Beamer, & Kornman, 1993). Scholars generally view PIM as a precursor of peri-implantitis (Lang & Berglunch, 2011). Therefore, prevention and management of PIM are essential in the long-term management of dental implants.

In animal and clinical studies, biofilm deposition on the implant surface has been identified as animportant etiological factor in initiating and developing peri-implant inflammation (Salvi, Aglietta, Eick, Sculean, Lang, & Ramseier, 2012). Several studies investigating the biofilm-related etiology of peri-implant inflammation have identified plaque removal as an essential step in resolving PIM (Ji, Tang, Wang, Cao, Cao, & Jin, 2014). Therefore, plaque removal from the peri-implant sites is considered critical in the long-term management of dental implants (Meijer, Raghoebar, Goene, & van der Weijden, 2011). Physical oral hygiene practices that remove dental plaque, such as toothbrushing (Huurzeler, Quinones, Schupback, Morrison, & Caffesse, 1997) or oral detergent (Schou, Berglundh, & Lang, 2004), should be prioritized for plaque removal. A single or combination treatment of systemic antibiotics, such as penicillin and metronidazole, may be used five to 14 days after the practice (Heitz–Mayfield & Lang, 2004). Furthermore, 0.12% chlorhexidine can be used for oral cavity disinfection (Schou, Berglundh, & Lang, 2004). Surgical treatments include removing bacteria and their by-products, calculus, and soft tissues attached to the implant's surface with surrounding bone loss, which creates an environment for bone re-adhesion (Baron, Haas, Dortbudak, & Watzek, 2000). Therefore, dental plaque management is essential in preventing and treating PIM.

The toothpick method (TPM), in which an expert directly performs Watanabe toothbrushing using a double-row type medium bristle toothbrush, is an effective method for dental plaque removal (Jang, 2001) that efficiently prevents and improves PIM (Park & Han, 2012). Chlorhexidine is effective when combined with TPM to treat PIM; its usage is gradually increasing (Trejo, Bonaventura, Weng, Caffesse, Bragger, & Lang, 2006). However, prolonged usage of chlorhexidine stains the teeth and gingiva, changes taste perception, and increases supragingival calculus (Russell & Day, 1993). Furthermore, it can cause adverse epitheliolysis of oral mucosa when used in pediatric patients. Moreover, adverse cases of shock, hypersensitivity, and temporary parotitis have also been reported for the drug (Brookes, Bescos, Belfield, Ali, & Roberts, 2020). Additionally, when chlorhexidine is used in combination with other drugs (including toothpaste), cationic component biding can reduce its efficacy (Haps et al., 2008). Thus, there is an increasing interest in replacing such chemical ingredients with natural substances showing bacterial inhibitory effects. Various studies have been conducted in this regard (S. Y. Lee et al., 2009).

Natural substances with demonstrated therapeutic effects for PIM include *Plectranthus scutellarioides* (L.) R. Br. Leaves extract (Bismelah, Ahmad, Mohamed Kassim, Ismail, & Rasol, 2022) and *Robusta Green Coffee Bean* (*Coffea Canephora*) (Nugraha et al., 2022). Often used in traditional Asian medicine, CB has been reported to have bioactive properties, such as anti-inflammatory effects in cells and bones. Various studies have been conducted to investigate CB's therapeutic properties: antioxidation (Ryu & Lee, 2008), anti-inflammation (J. Y. Lee et al., 2010), hemostasis (post-tooth extraction) (Zhou, 1985), regeneration and recovery of nerve cells (Kim, Han, Son, Jang, Kim, & Sin, 2022), and osteoclast formation inhibition (Cuong, 2009). However, no study has been conducted to investigate the combinatory effects of TBM and CB for managing PIM. Therefore, we investigated the antibacterial properties of the natural extract of CB against PIM-related bacteria in PIM patients subjected to TBM. Furthermore, CB was compared against chlorhexidine and saline, which are frequently used with TPM for implant patients in the clinic, to demonstrate the utility of the substance.

References

Ainamo, J., Barmes, D., Beagrie, G., Cutress, T., Martin, J., & Sardo–Infirri, J. (1982). Development of the World Health Organization (WHO) community periodontal index of treatment needs (CPITN). *Int. Dent. J., 32,* 281–291.

American Dental Association & American Academy of Periodontology. (2001). *Periodontal screening and recording—An early detection system* (pp. 1–8). Chicago: American Dental Association.

Baron, M., Haas, R., Dortbudak, O., & Watzek, G. (2000). Experimentally induced peri-implantitis: A review of different treatment methods described in the literature. *Int. J. Oral Maxillofac. Implants, 15*, 533–544.

Berglundh, T., Persson, L., & Klinge, B. (2002). A systematic review of the incidence of biological and technical complications in implant dentistry reported in prospective longitudinal studies of at least 5 years, discussion 232-3. *J. Clin. Periodontol., 29*(3), 197–212.

Bismelah, N. A., Ahmad, R., Mohamed Kassim, Z. H., Ismail, N. H., & Rasol, N. E. (2022). The antibacterial effect of Plectranthus scutellarioides (L.) R.Br. leaves extract against bacteria associated with peri-implantitis. *Journal of Traditional and Complementary Medicine 12*, 556e566. https://doi.org/10.1016/j.jtcme.2022.07.002.

Brookes, Z. L. S., Bescos, R., Belfield, L. A., Ali, K., & Roberts, A. (2020). Current uses of chlorhexidine for management of oral disease: A narrative review. *J. Dent., 103*, 103497. https://doi.org/10.1111/cod.13906.

Charalampakis, G., Jansåker, E., & Jansåker, A. M. R. (2014). Definition and prevalence of peri implantitis. *Curr. Oral Health Rep., 1,* 239–250.

Cheung, M. C., Hopcraft, M. S. & Darby, I. B. (2021). Dentists' preferences in implant maintenance and hygiene instruction. *Australian Dental Journal 66*(3), 278–288. https://doi.org/10.1111/adj.12831.

Christodoulides, N., Floriano, P. N., Miller, C. S., Ebersole, J. L., Mohanty, S., Dharshan, P., Griffin, M., Lennart, A., Ballard, K. L., King, C. P., Jr, Langub, M. C., Kryscio, R. J., Thomas, M. V., & McDevitt, J. T. (2007). Lab-on-a-chip methods for point-of-care measurements of salivary biomarkers of periodontitis. *Annals of the New York Academy of Sciences, 1098*(1), 411–428.

Cuong, N. X., Minh, C. V., Kiem, P. V., Huong, H. T., Ban, N. K., Nheim, N. X. & Kim, S. (2009). Inhibitors of steoclast formation from rhizomes of cibotium barometz. *J. Nat. Prod. 72*(9), 1673–1677

Derks, J., & Tomasi C. (2015). Peri-implant health and disease. A systematic review of current epidemiology. *J. Clin. Periodontol. 42*(|S16):S158–71.

Faria, G., Cardoso, C. R. B., Larson, R. E., Silva, J. S., & Rossi, M. A. (2009). Chlorhexidine- induced apoptosis or necrosis in L929 fibroblasts: A role for endoplasmic reticulum stress. *Toxicol. Appl. Pharmacol., 234*(2), 256–265.

Haps, S., Slot, D. E., Berchier, C. E., & Van der, Weijden, G. A. (2008). The effect of cetylpyridinium chloride-containing mouth rinses as adjuncts to toothbrushing on plaque and parameters of gingival inflammation: A systematic review. *Int. J. Dent. Hyg. 6*, 290–303.

Heitz–Mayfield, L. J., & Lang N. P. (2004). Antimicrobial treatment of peri-implant diseases *Int. J. Oral Maxillofac. Implants 19*, 128-139.

Hultin, M., Gustafsson, A., Hallstrom, H,. Johansson, L. A., Ekfeldt, A., & Klinge, B. (2002). Microbiological findings and host response in patients with peri-implantitis. *Clin. Oral Implants Res. 13*, 349–358.

Huurzeler, M. B., Quinones, C. R., Schupback, P., Morrison, E. C., & Caffesse, R. G. (1997). Treatment of peri-implantitis using guided bone regeneration and bone grafts, alone or in combination, in beagle dogs. Part 2: histologic findings. *Int. J. Oral Maxillofac. Implants 12*, 168–175.

Jang, G. W. (2001). Dental plaque control by professional toothbrushing. *Korean Dent. Assoc., 39*, 443-449

Ji, Y. J., Tang, Z. H., Wang, R., Cao, J., Cao, C. F., & Jin L. J. (2014). Effect of glycine powder air-polishing as an adjunct in the treatment of peri-implant mucositis: A pilot clinical trial. *Clin. Oral Implants Res., 25*(6): 683–689.

Kim, H. J., Kim, K-H., Lee, Y-M., Ku, Y., Rhyu, I-C., & Seol, Y-J. (2018). Peri-implant disease: Etiological factors and microbiological approaches. *Implantology, 22*(3), 174–182. https://doi.org/10.32542/implantology.20180015.

Kim, S.T., Han, Y. G., Son, H. S., Jang, S. J., Kim, J. S., & Sin, X. (2022). Isolation of the efficacy constituent for neuronal regeneration from cibotium barometz. *Yakhak Hoeji, 46*, 398–404.

Landry, R. G., & Jean, M. (2002). Periodontal screening and recording (PSR) index: Precursors, utility and limitations in a clinical setting. *Int. Dent. J., 52,* 35–40.

Lang, N. P., & Berglundh, T. (2011). Working Group 4 of Seventh European Workshop on Periodontology. Peri-implant diseases: Where are we now? Consensus of the Seventh European Workshop on Periodontology. *J. Clin. Periodontol., 38*(Suppl 11), 178–181.

Lang, N.P., Berglundh, T., Heitz–Mayfield, L. J., Pjetursson, B. E., Salvi, G. E., & Sanz M. (2004). Consensus statements and recommended clinical procedures regarding implant survival and complications. *Int. J. Oral Maxillofac Implants, 9*(Suppl): 150–154.

Lang, N. P., Brägger, U., Walther, D., Beamer, B., & Kornman, K. S. (1993) Ligature-induced peri-implant infection in cynomolgus monkeys. I. Clinical and radiographic findings. *Clin. Oral Implants Res., 4*, 2–11.

Lee, J. Y., Ko, S. H., Lee, Y. J., Lee, S. Y., Park, H. J., Shin, T. Y., & Jeon, H. I. (2010). Anti-inflammatory effect of MeOH extract of cibotium barometz in IFN-γ and LPS-stimulated mouse peritoneal macrophage. *Korean J. Pharmacogn., 41*(2), 108-114.

Lee, J. W., & Kim, M. B. (2016). *Composition and detection method for simultaneous detection of multiple oral disease-causing bacteria using multiplex real-time PCR.* KR Patent 10-1706070, filed July 25, 2016, and issued February 7, 2017.

Lee, S. Y., Kim, J. G., Baik, B. J., Yang, Y. M., Lee, K. Y., Lee, Y. H., & Kim, M. A. (2009). Antimicrobial effect of essential oils on oral bacteria. *J. Kor. Acad. Ped. Dent., 36,* 1–11.

Linkevicius, T., Puisys, A., Vindasiute, E., Linkeviciene, L. & Apse, P. (2013). Does residual cement around implant-supported restorations cause peri-implant disease? A retrospective case analysis. *Clin. Oral Implants Res. 24,* 1179–1184.

Meijer, H. J. A., Raghoebar, G. M., Goene, R. J., & van der Weijden, G. A. (2011). Complications in patients with oral implants. Recommendations for routine preventive inspections. *Nederlands Tijdschrift voor Tandheelkunde 118*, 431–437.

Mombelli, A., van Oosten, M. A., Schurch, E. Jr., & Land, N. P. (1987). The microbiota associated with successful or failing osseointegrated titanium implants. *Oral Microbiol. Immunol. 2*, 145–151.

Nomura, Y., Tamaki, Y., Eto, A., Kakuta, E., Ogino, D., Nakamura, Y., Takahashi, No., Hino, F., Koresawa, K., Hanada, N. & Shimizu, K. (2012). Screening for periodontal diseases using salivary lactate dehydrogenase, hemoglobin level, and statistical modeling. *Journal of Dental Sciences, 7*(4), 379–383.

Nugraha, A. P., Ardani, I. G. A. W., Sitalaksmi, R. M., Ramadhani, N. F., Rachmayanti, D., Kumala, D., Kharisma, V. D., Rahmadani, D., Puspitaningrum, M. S., Rizquianti, Y., Ari, M. D. A., Nugraha, A. P., Binti, T. N. E., Noor, T. A., & Luthfi, M. (2022). Anti-peri-implantitis bacteria's ability of robusta green coffee bean (coffea canephora) ethanol extract: An in silico and in vitro study. *Eur. J. Dent., 8.* doi:10.1055/s-0042-1750803.

Park, K. H., & Han, G. S. (2012). The effects of professional tooth cleaning and plaque control instruction on reduction of peri-implantitis. *J. Dent. Hyg. Sci., 12*, 173–180.

Park, K-H, Kim, Y-S., Lee, S-M., & Han, G-S. (2014). Willingness to pay for professional tooth cleaning in implant patients. *J. Dent. Hyg. Sci., 14*, 2, 176–182.

Persson, G. R., & Renvert, S. (2014). Cluster of bacteria associated with peri-implantitis. *Clin. Implant Dent. Relat. Res., 16*, 783–793.

Russell, A. D., & Day, M. J. (1993). Antibacterial activity of chlorhexidine. *J. Hosp. Infect. 25*, 229–238.

Ryu, M., & Lee, I. (2008). Antioxidant constituents from therhizomes of cibotium barometz. *Planta. Medica., 74,* 221.

Salvi, G. E., Aglietta, M., Eick, S., Sculean, A., Lang, N. P., & Ramseier, C. A. (2012). Reversibility of experimental peri-implant mucositis compared with experimental gingivitis in humans. *Clin. Oral Implants Res., 23,* 182–190.

Schou, S., Berglundh, T., & Lang, N. P. (2004). Surgical treatment of periimplantitis. *Int. J. Oral Maxillofac. Implants, 19*, 140–149.

Shibli, J. A., Melo, L., Ferrari, D. S., Figueiredo, L. C., Faveri, M., & Feres, M . (2008). Composition of supra- and subgingival biofilm of subjects with healthy and diseased implants. *Clin. Oral Implants Res., 19,* 975–982.

Trejo, P. M., Bonaventura, G., Weng, D., Caffesse, R. G., Bragger, U., & Lang, N. P. (2006). Effect of mechanical and antiseptic therapy on peri-implant mucositis: An experimental study in monkeys. *Clin. Oral Implants Res., 3*, 294–304. doi: 10.1111/j.1600-0501.2005.01226.x.

Zhou, R. J. (1985). Prevention and treatment of hemorrhage after tooth extraction by using the dry alum powder of cibotium barometz. *Zhong Xi Yi Jie He Za Zhi., 5*(8), 483-484, 452.

Zitzmann, N. U., & Berglundh, T. (2008) Definition and prevalence of peri-implant diseases. *J. Clin. Periodontol., 35* (Supplementum 8), 286–291.

Study goals and objectives

The purpose of this study was to confirm the improvement of peri-implant mucositis (PIM) in patients using the toothpick method (TPM) containing a natural ingredient: *Cibotium barometz J. Smith* (CB).

Study design

1. Type of study: A randomized, double-blind, controlled trial was conducted. In this study

2. Study population or sampling frame: The number of participants was determined using the G*Power 3.1 program based on significance level a = 0.05 bilateral test, power = 0.95, and effect size = 0.5. The result indicated that 54 participants were required for the study. Prior to the study, the purpose, procedures, and risks of the study were explained to the participants. The participants were informed that they had the right to discontinue the study at any stage before the consent was given. Initially, there were 72 participants recruited for the study. After excluding five participants who declined to participate or did not meet the inclusion criteria, 67 participants were selected. These participants were then randomly divided into three groups according to the solutions. The participants who showed abnormal data were excluded from the study, ultimately resulting in 60 participants. There were 19 participants in the saline-TPM group (SG), 20 in the chlorhexidine-TPM group (CG), and 21 in the Cibotium barometz J. Smith-TPM group (CBG) (Fig. 1).

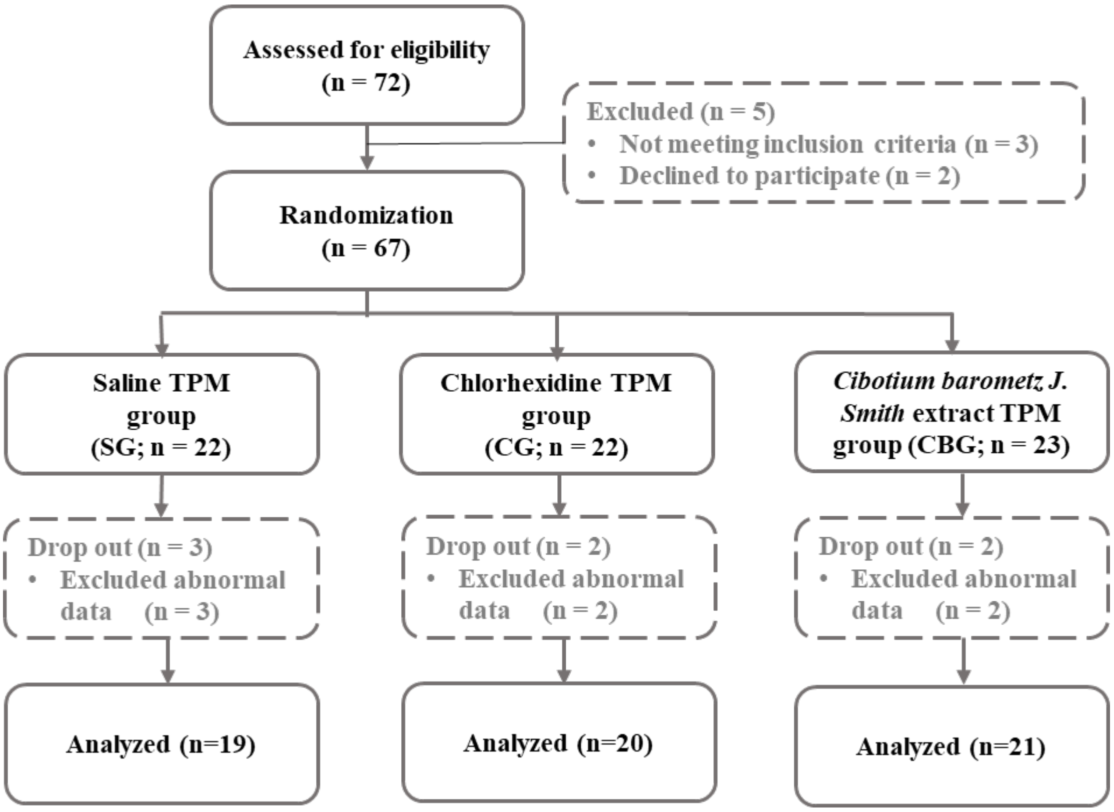

Fig. 1. Flow chart of study

3. Target Inclusion Criteria: Participants in this study were those who were not included in the exclusion criteria among the patients who agreed to fill out the questionnaire and had 16 or more remaining teeth and met the selection criteria.
4. Criteria for exclusion: The exclusion criteria were as follows: having serious dental caries with at least one dentin caries, xerostomia, smoking habit, chronic diseases, current antibiotic treatment, severe periodontal diseases with at least one tooth with a periodontal pocket depth of more than 4 mm, periodontal surgery with gum incision within the past month, or bleeding disorders.
5. Information on the expected study duration: As a randomized blind, controlled trial study, the study participants were selected among the patients of "M Dental Clinic" located in Busan, South Korea between January and July 2023. A dental hygienist with more than ten years of experience explained the purpose of this clinical trial, and only those who gave consent were included in the study. The selection criteria for the subjects were as follows: over 20 years old, having at least one oral implant, and having been diagnosed with PIM within the past year.

Methodology

To ensure homogeneity in the oral environment among the participants, a light scaling was performed by a trained dental hygienist after an oral examination by a dentist. The study commenced after one week of recovery. To confirm the oral health status, clinical indicators were assessed by examining the

periodontal treatment requirement, gingival bleeding, number of implants, and signs of oral examination. As for the periodontal disease-related factors, saliva analysis and changes in 12 types of PIM-related bacteria were evaluated. TPM was performed using a double-row medium bristle toothbrush applied with 10 mg/ml CB extract (CBG), 0.12% chlorhexidine (CG), or saline (SG). After the scaling (one week before the study commencement), toothbrushes and toothpaste were provided to the participants. The participants were trained on the method and frequency of tooth brushing needed for the study. For all three groups, the clinical indicators related to the periodontal status were collected both before and after TPM (Fig. 2).

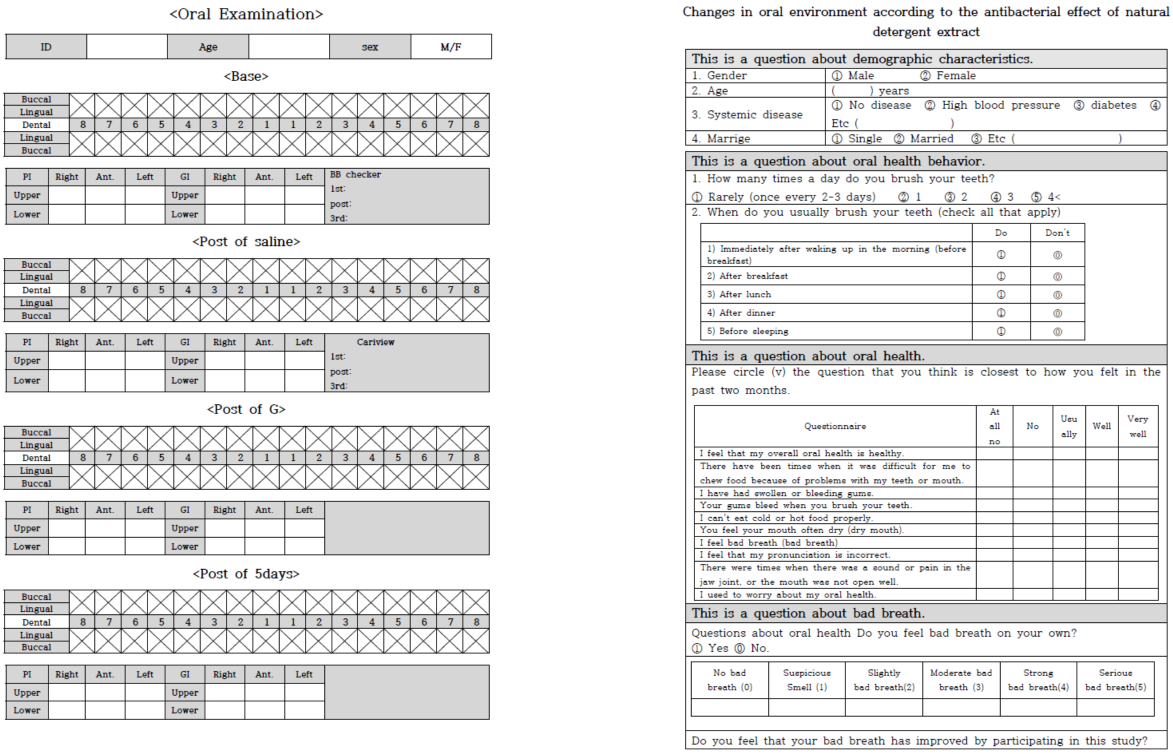

<Oral Examination>

ID		Age		sex	M/F

<Base>

Buccal																
Lingual																
Dental	8	7	6	5	4	3	2	1	1	2	3	4	5	6	7	8
Lingual																
Buccal																

PI	Right	Ant.	Left	GI	Right	Ant.	Left	BB checker
Upper				Upper				1st: post: 3rd:
Lower				Lower				

<Post of saline>

Buccal																
Lingual																
Dental	8	7	6	5	4	3	2	1	1	2	3	4	5	6	7	8
Lingual																
Buccal																

PI	Right	Ant.	Left	GI	Right	Ant.	Left	Cariview
Upper				Upper				1st: post: 3rd:
Lower				Lower				

<Post of G>

Buccal																
Lingual																
Dental	8	7	6	5	4	3	2	1	1	2	3	4	5	6	7	8
Lingual																
Buccal																

PI	Right	Ant.	Left	GI	Right	Ant.	Left	
Upper				Upper				
Lower				Lower				

<Post of 5days>

Buccal																
Lingual																
Dental	8	7	6	5	4	3	2	1	1	2	3	4	5	6	7	8
Lingual																
Buccal																

PI	Right	Ant.	Left	GI	Right	Ant.	Left	
Upper				Upper				
Lower				Lower				

Changes in oral environment according to the antibacterial effect of natural detergent extract

This is a question about demographic characteristics.	
1. Gender	① Male ② Female
2. Age	( ) years
3. Systemic disease	① No disease ② High blood pressure ③ diabetes ④ Etc ( )
4. Marrige	① Single ② Married ③ Etc ( )

This is a question about oral health behavior.

1. How many times a day do you brush your teeth?
① Rarely (once every 2-3 days) ② 1 ③ 2 ④ 3 ⑤ 4<

2. When do you usually brush your teeth (check all that apply)

	Do	Don't
1) Immediately after waking up in the morning (before breakfast)	①	⓪
2) After breakfast	①	⓪
3) After lunch	①	⓪
4) After dinner	①	⓪
5) Before sleeping	①	⓪

This is a question about oral health.

Please circle (v) the question that you think is closest to how you felt in the past two months.

Questionnaire	At all no	No	Usu ally	Well	Very well
I feel that my overall oral health is healthy.					
There have been times when it was difficult for me to chew food because of problems with my teeth or mouth.					
I have had swollen or bleeding gums.					
Your gums bleed when you brush your teeth.					
I can't eat cold or hot food properly.					
You feel your mouth often dry (dry mouth).					
I feel bad breath (bad breath)					
I feel that my pronunciation is incorrect.					
There were times when there was a sound or pain in the jaw joint, or the mouth was not open well.					
I used to worry about my oral health.					

This is a question about bad breath.

Questions about oral health Do you feel bad breath on your own?
① Yes ⓪ No.

No bad breath (0)	Suspicious Smell (1)	Slightly bad breath(2)	Moderate bad breath (3)	Strong bad breath(4)	Serious bad breath(5)

Do you feel that your bad breath has improved by participating in this study?

Fig. 2. Clinical chart

1. Clinical examination

1.1. Oral Examination, Number of Implants, Subject Satisfaction.

To investigate the oral health status of the participants, the signs of regular oral examination and the number of implants in the oral cavity were investigated. Furthermore, the level of satisfaction after TPM was surveyed for all three groups.

1.2. Periodontal Screening and Recording (PSR).

The Periodontal Screening and Re-cording (PSR) is a modification of the Community Periodontal Index of Treatment Needs (CPITN), which was developed by the World Health Organization to estimate

periodontal treatment needs in population groups (Ainamo, Barmes, Beagrie, Cutress, Martin, & Sardo–Infirri, 1982).With PSR, a specialized periodontal probe with a ball-shaped tip having a 0.5-mm diameter isused to examine and score six sites per tooth in each patient dentition sextant on a 0 to 4 hierarchical grading scale, identical to CPITN criteria,6with only the highest PSR score per sextant record for documentation. Sextants with only shallow probing depths (PDs) <3.5 mm at all tooth sites are assigned a PSR index score of either 0, 1, or 2, depending on presence of bleeding on probing (BOP), dental calculus deposits, and/or defective dental restoration margins in the sextant. Sextants with deepest PDs ranging be-tween 3.5 and 5.5 mm are assigned a PSR index score of 3, whereas sextants with at least one>5.5-mm PD receive a PSR index score of 4. In addition to the 0 to 4 grading scale, a code is to be added to PSR scores when additional periodontal abnormalities are detected in a dentition sextant, such as furcation involvements, excessive tooth mobility, mucogingival problems, and recession defects of ≥3.5 mm (American Dental Association & American Academy of Periodontology, 2001; Landry & Jean, 2002).

1.3. Bleeding of probing (BOP).

BOP was measured using a standardized periodontal probe (CP11, Hu-Friedy Mfg. Co., LLC., Chicago, Illinois, USA) with 20-25g probing force. The cases of bleeding within 30 seconds of probing the buccal and lingual surface of each tooth were recorded as a percentage. To determine the accuracy in measuring bleeding, the measurements were taken from the most posterior molar.

1.4. Saliva Test Analysis System.

According to the instructions provided for Sill-Ha ST-4910 (Arkray Inc., Kyoto, Japan), the subjects rinsed their mouth with 3 mL of mouthwash for 10 seconds prior to spiting the saliva out into sterile tubes. The saliva was collected from the tube with a dropper and then placed on a measuring strip, which was then mounted on a measurement strip holder. Periodontal health (occult blood, leukocytes, proteins) was analyzed after 5 minutes and documented. The average values provided by the manufacturer were used as standard: average blood count = 22, average leucocyte count = 49, and average protein count = 43. It was evaluated that higher counts correlated with poorer periodontal health.

1.5. Microbiological Analysis of PIM.

To collect subgingival microbial samples, sterilized paper points (size #15) were inserted in two maxillas (#16 and #21) and two mandibles (#36 and #41) for ten seconds before placing them in sterilized 1.5 mL tubes. Next, DNA was extracted from the samples using the AccuPrep Universal RNA Extraction Kit (Bioneer, Daejeon, South Korea) for microbial analysis. According to the manufacturer's instruction, OligoMix (YD Global Life Science Co., Ltd., Seongnam, South Korea) and three

oligonucleotides (forward primer, reverse primer, and probe), which react specifically to each bacterium, were used (Lee & Kim, 2016, 2017).

The 12 types of PIM-related bacteria (Table 1) were as follows: *Parvimonas micra (P. micra), Eubacterium nodatumm (E. nodatumm), Porphyromonas gingivalis (P. gingivalis), Tannerella forsythia (T. forsythia), Treponema denticola (T. denticola), Fusobacterium nucleatum (F. nucleatum), Prevotella intermedia (P. intermedia), Prevotella nigrescens (P. nigrescens), Eikenella corrodens (E. corrodens), Campylobacter rectus (C. rectus), Aggregatibacter actinomycetemcomitans (A. actinomycetemcomitans), Staphylococcus aureus (S. aureus)* (Kim, Kim, Lee, Ku, Rhyu, & Seol, 2018).

First, 9 μL of OligoMix, 10 μL of 2x probe qPCR mix (Takara Bio Inc., Shiga, Japan), and 1 μL of template DNA were mixed. Next, the polymerase chain reaction (PCR) reaction samples were placed in a 96-well plate, which was installed in the CFX96 Touch Real-Time PCR Detection System (Bio-Rad, Hercules, USA) for DNA segment amplification. The cycle condition of PCR was as follows: PCR initial activation step at 95 °C for 30 seconds, denaturation for 10 seconds at 95 °C, and annealing at 62 °C for 30 seconds with 40 repeated cycles. The result was then analyzed as cycle threshold (Ct) using Bio-Rad CFX Manager Software.

Table 1. Primers and probes used in the real-time PCR assays

**Bacteria**	**Target genes**	**Primers/Probe sets**	**Amplicon size (bp)**
Parvimonas micra	16S ribosomal RNA gene	5′-GAGGAATACCGGTGGCGAAG-3’<br>5′-GGCACCGAGATTTGACTCCC-3’<br>5′-FAM-GGTACGAAAGCGTGGGGAGCA-BHQ1–3’	148
Eubacterium nodatum	hypothetical protein	5′-TGCTTGCCGGTGACTTAGGA-3’<br>5′-AAACCGGGCTCAACAACCAT-3’<br>5′-Texas Red-TTGAGGAGCCGGTGACTTTGG-BHQ2–3’	130
Porphyromonas gingivalis	hemagglutinin (phg) gene	5′-ACACGGTGTATCGTGACGGC-3’<br>5′-GCCGGCTGCGTACTTAACCT-3’<br>5′-HEX-CGACCTACCGCGATGCAGGA-BHQ1–3’	119
Tannerella forsythia	karilysin protease gene	5′-TGGCAAATCGCTCATCATCC-3’<br>5′-TTCCATGTTCCCCAACCACA-3’<br>5′-Texas Red-CCATTAAGCCCATTGCCCGG-BHQ2–3’	140
Treponema denticola	oligopeptidase B (opdB) gene	5′-AGAAAGGCTTTGGGCGACAG-3’<br>5′-GCTGGAGCCGTAGCTTCCAT-3’<br>5′-Cy5-CGGGTCCTCACCCGCTCTTC-BHQ2–3’	127
Fusobacterium nucleatum	16S ribosomal RNA gene	5′-GGCTGTCGTCAGCTCGTGTC-3’<br>5′-CTCATCGCAGGCAGTATCGC-3’<br>5′-FAM-AACGAGCGCAACCCCTTTCG-BHQ1–3’	114
Prevotella intermedia	hemagglutinin (phg) gene	5′-CACACGCTGGCGAAACCTAC-3’<br>5′-CACGTGGCGTTGCTTCTTTC-3’<br>5′-HEX-CCGAAGATGCGCCGTTGAAC-BHQ1–3’	143
Prevotella nigrescens	gyrase subunit B (gyrB) gene	5′-AGCAAGCTGTAGGCGAGGCT-3’<br>5′-GCTGAACACTTTCGCGTGCT-3’<br>5′-Texas Red-GCTCGTATTGCAGCCCGCAA-BHQ2–3’	132

Bacteria	Target genes	Primers/Probe sets	Amplicon size (bp)
Eikenella corrodens	proline iminopeptidase (pip) gene	5′-GCCAACTGCTGCTGGAAGTG-3’<br>5′-GCCGCTGATTTCGGAGAGTT-3’<br>5′-HEX- ACAGCCATCGGCACAGGCAT-BHQ1–3’	110
Campylobacter rectus	groEL gene	5′-AAATTTAAGCGGCGACGAGG-3’<br>5′-TCCTTGCTCACGCTTACGGA-3’<br>5′-HEX-GGCTTTGACGCGGGCGTAGT-BHQ1–3’	132
Aggregatibacter actinomycetemcomitans	leukotoxin gene	5′-CGGGGCTTTCTACTACGGGA-3′<br>5′-ATGCCTCAAGCATTCTCGCA-3′<br>5′-FAM-GGTCAGCTTGGCAATCAGCCC-BHQ1–3’	123
Staphylococcus aureus	clumping factor A (clfA) gene	5′-GCGCAAGTAACGAAAGCAAAA-3’<br>5′-GATTTTGCGCCACACTCGTT-3’<br>5′-FAM-TGCTGCACCTAAAACAGACGACACA-BHQ1–3’	132

Safety considerations

The investigator explained safety considerations to the subjects before the study began.

[It is judged that there will be no side effects that may appear in this study, and if there is a subject who occasionally shows an adverse reaction during the oral examination, a list of the relevant oral examinations will be selected and excluded from the data collection stage. In addition, in this study, subjects who quit midway during the oral examination process will be excluded from the subject.]

Follow up

The investigator explained the follow-up to the subjects before the study started.

[If a health problem occurs due to an adverse reaction, all treatment costs are borne by the researcher, and the patient's health is protected until the side effect is resolved by performing follow-up.]

Data management and statistical analysis

All obtained clinical results were analyzed at a significance level of 5% using SPSS 24.0 for Windows (IBM Corp., Armonk, NY, USA). To confirm the difference in demographic characteristics and periodontal-related clinical indicators between SC, CG, and CBG, ANOVA and chi-square tests were conducted. Paired t-test was conducted to verify the differences in indicators between pre- and post-TPM for all three groups. Furthermore, ANOVA was conducted to evaluate the average differences in saliva tests and PIM-related bacteria levels between the three groups. Duncan’s post-hoc test was performed for post hoc analysis.

Quality assurance

The researcher conducted the study after sufficiently explaining the overall process of the study to the

subject.

[If you indicate your intention to participate, the following process will proceed. You will receive a three-minute questionnaire at Busan M Dental Clinic. After filling out simple personal information about gender and age, you will be given an oral examination. Periodontal examination and microbial activity test are performed as oral examination items, and it takes about 30 minutes. You may withdraw from participation at any time and without any penalty. If you wish to stop participating in the study, please inform the researcher immediately.

In addition, we will promptly destroy data collected in the study when you discontinue participation.]

Expected outcomes of the study

As part of our interest in new, next-generation antibacterial materials, we would like to confirm the value of its use as an antibacterial agent against bacteria that cause peri-implantitis in the oral cavity and suggest the possibility of its use as a gargle to improve implant maintenance.

Dissemination of results and publication policy

This study recorded and distributed their current oral health status to the study subjects. The management method was explained according to the oral health status, and the results of the oral status were analyzed only by those who consented to the study and submitted to the journal because the subject's identifier was removed.

Duration of the project

1. Data collection: About one month
2. Data analysis: expected to take about one month
3. Organize data: It will take about three months

If the study subjects are not recruited due to COVID-19, the data collection period may be longer than expected. As a countermeasure, the data collection period was reset to two months.

Project management

Prof. Seol-hee Nam is responsible for this research, and Professor Seol-hee Nam conducted and managed all processes and explained this to the research subjects in advance.

[The person in charge of personal information management is Professor Seoul-Hee Nam of Kangwon National University, 033-540-3394. We will do our best to ensure the confidentiality of any personal information obtained through this research. Your name and other personal information will not be used when the personal information obtained from this research is disclosed in academic journals or conferences. However, if required by law, your personal information may be provided. In addition, monitor personnel, inspectors, and bioethics committees can directly view the research results to

verify the reliability of the research's procedures and data within the scope of the relevant regulations without infringing on the confidentiality of the study participants' personal information. By signing this consent form, you agree that you have been informed of and agree to do so.]

Ethics

The researcher explained research ethics to the subjects before the study started.

[To protect the information of research subjects, unnecessary personal identifiers of collected data are removed. In particular, do not write the subject's name, resident registration number, etc. in the demographic characteristics question, and the identifier code linked to the personal information is managed separately. In addition, when presenting the research results, it should be presented in a form that cannot confirm the identity of the individual. After the completion of the study, research-related data will be stored in an enclosed space (cabinet) in the laboratory of the principal investigator for 3 years, and the information stored in the personal laptop will be 'permanently deleted'. Documented research documents will be destroyed so that personal information cannot be distinguished through a shredder after submitting a report to the host organization of this research and submitting a thesis. Data and personal information of subjects whose participation in the study has been suspended or withdrawn will be destroyed immediately.]

Informed consent forms

The consent form is attached as file #2.

References

Lee, J. Y., Ko, S. H., Lee, Y. J., Lee, S. Y., Park, H. J., Shin, T. Y., & Jeon, H. I. (2010). Anti-inflammatory effect of MeOH extract of cibotium barometz in IFN-γ and LPS-stimulated mouse peritoneal macrophage. *Korean J. Pharmacogn., 41*(2), 108-114.

Nomura, Y., Tamaki, Y., Eto, A., Kakuta, E., Ogino, D., Nakamura, Y., Takahashi, No., Hino, F., Koresawa, K., Hanada, N. & Shimizu, K. (2012). Screening for periodontal diseases using salivary lactate dehydrogenase, hemoglobin level, and statistical modeling. *Journal of Dental Sciences, 7*(4), 379–383.

Salvi, G. E., Aglietta, M., Eick, S., Sculean, A., Lang, N. P., & Ramseier, C. A. (2012). Reversibility of experimental peri-implant mucositis compared with experimental gingivitis in humans. *Clin. Oral Implants Res., 23,* 182–190.

Research protocol: part 2

Budget

The budget section should include a detailed, item-by-item breakdown of the funds requested, along with the rationale for each item.

1. *Cibotium Barometz J. Smith* extract

2. Clinical examination fee

Not applicable

Other support for the project

Name of the institution. : Not applicable

Types of organs. : Not applicable

Research project number. : Not applicable

Collaboration with other scientists or research institutions

Not applicable.

Curriculum Vitae of investigators

Researchers				
Research Manager	Name	affiliation	Position	A major field of study
	Seoul-Hee Nam	Department of Dental Hygiene, College of Health Sciences, Kangwon National University	Associate professor	Dentistry
Co-Researcher	Yu-Rin Kim	Department of Dental Hygiene, Silla University,	Assistant professor	Oral health

Other research activities of the investigators

Not applicable

Financing and insurance

Not applicable.
